# Anthrax: Public Health Risk in India and Socio-Environmental Determinants

**DOI:** 10.4103/0970-0218.62573

**Published:** 2010-01

**Authors:** Rajan R Patil

**Affiliations:** School of Public Health, SRM University, Kattankulathur, Chennai, India

## Introduction

Zoo authorities of Central Indian State of Chattisgarh reported a hyena suffering from the fatal anthrax disease in Nandanvan zoo in capital city Raipur. After laboratory confirmation of anthrax, the zoo was closed for 15 days as precautionary measure. According to the veterinarians of the zoo, all symptoms of anthrax were found in one of the hyenas. The Forest department officials decided to vaccinate animals within the 5 km radius of zoo to contain the disease. All employees of Nandanvan zoo had been put on prophylactic antibiotic medicine.(1)

The report of death of a hyena due to anthrax in the state of Chattisgarh in India during June 2007 was very disturbing and was unusual occurrence by any standards. Chattisgarh state has never reported anthrax case either in animal or in humans. Anthrax is generally an epizootic disease which infects and kills herbivorous animals e.g. cattle, sheep, and goat.(2) Cattle get mostly infected by eating vegetations contaminated by spores. Elephants have been known to die of anthrax in north-east. They and other herbivores develop bacteriamic phase followed by death. So do poor humans. Occasionally dogs, horses, etc. are also found to be infected but are rarely fatal in them. Terminally ill animals tend to bleed from the nose, mouth, and bowel, thereby contaminating soil or water places with B. anthracis that can subsequently sporulate and persist in the environment.

On further literature review, the Chattisgarh incident turns out to be the first case of death of non-herbivorous animal like hyena dying due to anthrax in India.(3) This event has a great epidemiological as well as public health significance. The reports of anthrax infection in hyena leading to death is a pointer towards the larger fact that the anthrax bacteria are in circulation in Chattisgarh, also it is a matter of concern from public health point of view, as once anthrax affected, the locale is always anthrax prone on account of longevity of viable spores lasting many decades.

From public health point of view, it is very important that Chattisgarh should step up their mechanism for animal screening for anthrax. Since Chattisgarh never had history of anthrax, capacities and skills for the same need to be instituted. Once anthrax is fully established as enzootic disease in a region, it is only a matter of time before zoonotic transmission begin and human case of anthrax begin to appear posing as big public health problem. The case in point is Orissa, where anthrax is an endemic disease and already a major public health problem of Orissa; 14 out of 30 revenue districts in the state have witnessed outbreaks of anthrax as many as 61 times during the last 10 years affecting 750 people of which 418 had died.(4) The anthrax outbreaks are an annual phenomenon Orissa. With good forest cover, the soil being highly organic in with good moisture to support anthrax spores. Due to scanty agriculture, tribals mainly depend on forest for livelihood. Risk of infection increases due to anthrax spores in the wild. Outbreaks of anthrax in these indigenous populations very often occur as food poisoning following consumption of contaminated cattle meat.

Tribals for that matter, any given community, in these states particularly if underprivileged, eat carcass of dead animals. Such people are vulnerable. Even if they do not eat, if they de-skin carcasses of infected animals, they are also vulnerable for anthrax infection.

Anthrax is known to occur generally in underdeveloped regions.(5) Ecologically speaking, from vulnerability point of view, Chattisgarh shows striking parallels and Orissa, as far as potential proneness and risk of anthrax outbreaks in the human population in the coming years:

1. Chattisgarh and Orissa rank almost similar on human development indices (HDIs);
2. Like Orissa, Chattisgarh has very high concentration of tribal population (>35%);
3. Both the states have extensive forest cover;
4. The indigenous population of both these states depend less on agriculture and more on forest and animal produce for food;
5. Overwhelming majority of people in both these states live below the poverty line.

Both the states have poor public health infrastructure. This is the perfect amalgamation and interface of risk factors that are conducive for zoonotic transmission of anthrax to the human population. Hence the probability of occurrence of anthrax outbreak in the human population would increase on logarithmic scale, each time an episode of anthrax detected in animal population in a state like Chattisgarh.

So, epizootology and epidemiology should be both applied not only for anthrax, but for every zoonotic disease, hence the importance of veterinary and doctors putting heads together.

## Public Health Action Recommend

1. Anthrax disease needs to be prevented with proper legislation for meat handling as well as effective immunization of animals.(6) Regulation of meat sale and good hygiene practices at slaughterhouses. Animals for human consumption should undergo inspection before they are slaughtered and put on sale.
2. Coordination with veterinary department and animal husbandry department and for surveillance and livestock vaccination drive for of all the cattle.
3. Behavioral change communication for anthrax prevention--all the hamlets educating the inhabitants with very important one line health education input. Not to consume raw meat, cook it well before eating else you may be at risk of contracting anthrax.
4. Inter-state cooperation between Chattisgarh and Orissa on ways on the management of anthrax prevention and control.
5. Orissa is carrying out annual vaccination drive of livestock in the affected districts like Koraput, Kalahandi, Malkangiri, etc. Chattisgarh state could find the sources for funding for this program in Orissa.
6. It is important to take up live stock vaccination against anthrax. It is one of the most credible way of providing health security to the vast majority of underprivileged sections of the society, to prevent them from further impoverishment by breaking the cycle of poverty and infection.
7. The actual incidence of anthrax in India is not known accurately mostly due to underreporting.(5) Hence every case suspected of anthrax infection/death should be reported. From this point of view, the isolated anthrax incident in hyena from Chattisgarh gains importance.
